# Memory control deficits in the sleep-deprived human brain

**DOI:** 10.1073/pnas.2400743122

**Published:** 2024-12-31

**Authors:** Marcus O. Harrington, Theodoros Karapanagiotidis, Lauryn Phillips, Jonathan Smallwood, Michael C. Anderson, Scott A. Cairney

**Affiliations:** ^a^Department of Psychology, University of York, York YO10 5DD, United Kingdom; ^b^School of Psychology, University of East Anglia, Norwich NR4 7TJ, United Kingdom; ^c^School of Psychology, University of Sussex, Brighton BN1 9QU, United Kingdom; ^d^Department of Psychology, Queen’s University, Kingston K7L 3N6, Canada; ^e^Medical Research Council Cognition and Brain Sciences Unit, University of Cambridge, Cambridge CB2 7EF, United Kingdom; ^f^York Biomedical Research Institute, University of York, York YO10 5NG, United Kingdom

**Keywords:** default mode network, heart rate variability, inhibitory control, memory suppression, sleep deprivation

## Abstract

Sleep disturbances are associated with intrusive memories, but the neurocognitive mechanisms underpinning this relationship are poorly understood. Here, we show that sleep deprivation disrupts prefrontal inhibition of memory retrieval, and that the overnight restoration of this inhibitory mechanism is associated with time spent in rapid eye movement (REM) sleep. The functional impairments arising from sleep deprivation are linked to a behavioral deficit in the ability to downregulate unwanted memories, and coincide with a deterioration of deliberate patterns of self-generated thought. We conclude that sleep deprivation gives rise to intrusive memories via the disruption of neural circuits governing mnemonic inhibitory control, which may rely on REM sleep.

## Significance

Sleep problems and intrusive memories play an important role in the onset and maintenance of many mental health disorders. Here, we show that depriving healthy participants of sleep disrupts their ability to keep intrusive memories at bay. Using functional neuroimaging, we reveal that these deficits are related to difficulties in engaging brain regions that support the inhibition of memory retrieval. These findings offer fresh insight into our understanding of the cognitive and neural mechanisms underlying the link between poor sleep and mental illness and could support the development of novel treatment and prevention strategies.

Memories of unpleasant experiences can intrude into conscious awareness, often in response to reminders. While such intrusive memories are an occasional and momentary disturbance for most people, they can be recurrent, vivid, and upsetting for individuals suffering from mental health disorders such as depression, anxiety, and posttraumatic stress disorder (1). Given the transdiagnostic significance of intrusive memories, a better understanding of the mechanisms that precipitate their occurrence is vital to improving emotional well-being and reducing the global burden of mental illness.

One way people ward off intrusive memories is by suppressing their retrieval, purging them from awareness. Direct suppression of unwanted memories serves an adaptive function in that it weakens the corresponding memory trace and thereby decreases the likelihood of future memory intrusions [(2–8), for review, see ref. 9]. In previous work, we showed that adaptive memory suppression is critically dependent on sleep (10). Whereas well-rested individuals could override retrieval operations and reduce the emergence of intrusive memory content in subsequent trials, sleep-deprived individuals failed to suppress the target memories effectively, which remained intrusive over time.

The adaptive benefits of memory suppression are associated with high-frequency heart rate variability (HF-HRV; 0.15 to 0.40 Hz)—a physiological correlate of cognitive control (11). Specifically, higher trait HF-HRV has been linked to better suppression-induced forgetting of unwanted memories (12) and larger suppression-related improvements in emotional reactivity to negative images (10). However, it has yet to be established whether trait HF-HRV is associated with people’s ability to downregulate unwanted memories over time. Moreover, whether the benefit of higher HF-HRV for memory suppression is affected by sleep deprivation remains an open question.

At the neurocognitive level, memory suppression is orchestrated by the right dorsolateral prefrontal cortex (rDLPFC), which, via top–down inhibitory modulation, downregulates retrieval operations in the hippocampus (2, 3, 13, 14), a process that relies on local GABAergic inhibition (15). We recently proposed that disruption to this memory control network by sleep deprivation can explain how recurrent failures of memory suppression arise after prolonged wakefulness (16). Indeed, rDLPFC is a domain-general inhibitory control region implicated in the stopping of actions as well as unwanted memories (13, 17); sleep deprivation reduces rDLPFC engagement during motor response inhibition, leading to a breakdown in task performance (18, 19). Relatedly, sleep deprivation impairs prefrontal control of the amygdala during threat-related information processing, prompting an overnight increase in state anxiety (20). Whether an absence of sleep impairs prefrontal inhibition of hippocampus during memory suppression has yet to be determined, but is key to understanding the neurocognitive mechanisms by which sleep deprivation gives rise to intrusive thoughts.

Suppressing unwanted memories in a goal-directed manner is also broadly dependent on the adaptive segregation of whole-brain functional networks. The default mode network (DMN) is a collection of interacting brain regions, including medial prefrontal and posterior cingulate cortex, that are commonly activated at rest (i.e., when focusing on one’s internal mental state) and deactivated during attention-demanding tasks (21, 22). The DMN is anticorrelated with the frontoparietal cognitive control network (CCN), a task-positive network encompassing lateral prefrontal and superior parietal areas (23). Supported by ascending arousal input from the thalamus, performance of externally driven, goal-directed tasks is thought to rely on disengagement of the DMN and reciprocal engagement of the CCN (24, 25). Inappropriate perseverance of the DMN during attention-demanding cognitive tasks, as well as unstable thalamic activity, are a common consequence of sleep deprivation (26, 27). Likewise, resting-state (RS) fMRI data derived from sleep-deprived individuals reveals a breakdown of functional decoupling between the DMN and CCN (23, 28) and aberrant connectivity between the DMN and thalamus (29). Inappropriate gating of on-task relative to off-task network activity, together with reduced ascending arousal input from the thalamus, may therefore contribute to failures of adaptive memory control when attempting to suppress the retrieval of internally generated information.

Disruption to the brain networks underlying dynamic cognitive control by sleep deprivation should manifest behaviorally not only in failures of adaptive memory suppression, but also in the content of self-generated thoughts. Specifically, an individual who cannot suppress unwanted thoughts effectively is likely to show a proclivity for unsolicited mental content and thus a reduction in deliberate and focused patterns of thinking. Indeed, when assessed in externally demanding task contexts, sleep-deprived individuals report a higher proportion of task-unrelated thoughts than well-rested individuals (30), signifying a breakdown of control processes that allocate attentional resources in accordance with environmental requirements. However, extant findings concerning the effects of sleep deprivation on self-generated mental content are based on the self-reported categorization of on-task and off-task thoughts. Whether phenomenological patterns of ongoing thought following sleep deprivation are consistent with the degradation of inhibitory memory control has yet to be established.

We sought to delineate the neurocognitive mechanisms through which sleep deprivation gives rise to intrusive memories and the associated consequences for ongoing patterns of self-generated thought. First, we tested the hypothesis that sleep deprivation impairs prefrontal inhibition of memory retrieval operations in the hippocampus. Participants suppressed emotionally negative and neutral memories while undergoing functional MRI (fMRI) after one night of total sleep deprivation or restful sleep. We tested the prediction that sleep deprivation weakens rDLPFC engagement during memory suppression, resulting in a concomitant increase in hippocampal activation and a failure to adaptively downregulate intrusive memories.

Given the deleterious effects of sleep deprivation on memory suppression, a reciprocal question concerns the specific components of sleep that underpin the overnight restoration of prefrontal mnemonic control. Rapid eye movement (REM) sleep is thought to play an important role in decreasing next-day brain reactivity to emotional experiences by supporting the top–down control of the amygdala by the prefrontal cortex [(31), for review see ref. 32]. REM sleep might, therefore, support the engagement of a similar top–down control process when confronted with reminders to unwanted memories. Indeed, individuals suffering from psychiatric disorders who have difficulty suppressing unsolicited thoughts often exhibit abnormalities of REM sleep, alongside other sleep alterations (33–38). We therefore recorded restful sleep with polysomnography (PSG) to determine the contributions of REM sleep to memory suppression processes governed by rDLPFC.

Because memory suppression is broadly reliant on the adaptive segregation of whole-brain networks governing internally focused mental processing and externally directed cognitive control, we also sought to confirm prior findings that sleep deprivation impairs functional decoupling between DMN and CCN and leads to unstable thalamic input to these regions. Sleep-rested and sleep-deprived participants thus completed a RS fMRI scan immediately after the memory suppression task, allowing us to test the prediction that sleep deprivation increases functional connectivity between off-task (DMN) and on-task (CCN) networks, and reduces thalamic connectivity with these networks.

Finally, we asked whether sleep deprivation reduces the occurrence of deliberate and focused self-generated thoughts, consistent with a breakdown of inhibitory memory control. Before and after the overnight delay, participants described the content of their ongoing thoughts as they engaged in either a cognitively demanding task that required engagement of working memory (1-back) or a nondemanding task (0-back), allowing us to test the prediction that sleep deprivation reduces patterns of deliberate, on-task thinking.

## Results

### Sleep Deprivation Impairs Prefrontal Memory Control.

**Fig. 1.**
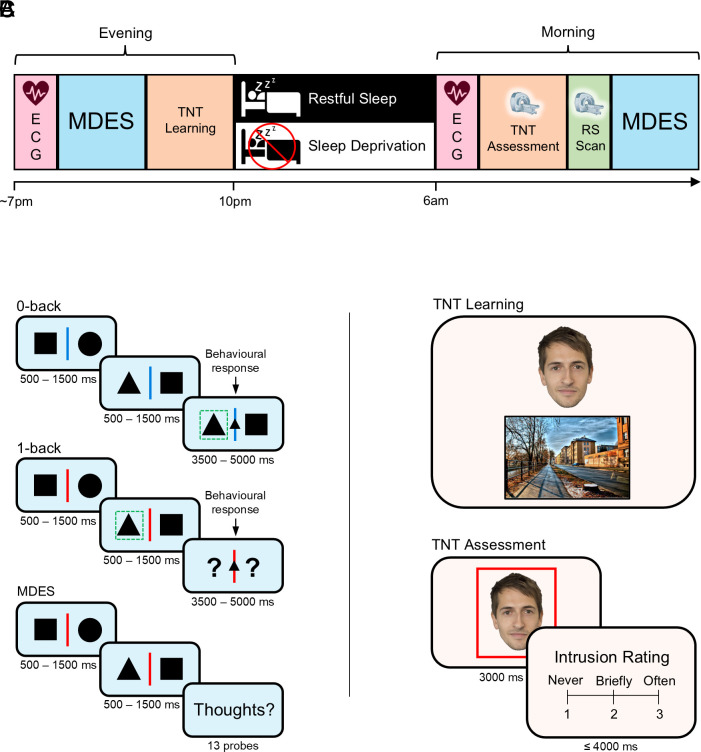
Experimental procedure. (*A*) Timeline. The evening session began with a resting electrocardiography (ECG) recording. Participants then completed the first of two multidimensional experience sampling (MDES) tasks and the TNT learning phase, before either sleeping overnight in the laboratory where their sleep was recorded with PSG (restful sleep group) or remaining awake throughout the night (sleep deprivation group). The morning session began with another resting ECG recording. Participants then completed the TNT assessment phase inside a MRI scanner, after which a RS scan was acquired. Finally, participants completed the MDES task again. (*B*) MDES task. Participants monitored pairs of shapes. After 2 to 5 of these nontarget trials, a target trial occurred, wherein an additional shape appeared in the center of the screen, prompting participants to press a button corresponding to the side of the screen that the matching shape appeared on the present trial (0-back) or the immediately preceding trial (1-back). Occasionally, instead of a target trial, participants were required to indicate via a rating scale (1 to 10) the extent to which the contents of their ongoing thoughts matched a series of 13 thought probes. (*C*) TNT task. In the TNT learning phase, participants memorized 48 face-scene pairs. In the TNT assessment phase, faces were shown in isolation inside red or green frames. For red-framed faces (No-Think trials), participants were instructed to suppress (i.e., avoid thinking about) the associated scene. For green-framed faces (Think trials), participants were instructed to visualize the associated scene. After each trial, participants reported the extent to which they thought about the paired scene (never, briefly, or often).

After a night of sleep deprivation (n = 43; 18 male; mean ± SD age: 19.58 ± 1.72 y) or restful sleep (n = 42; 12 male; mean ± SD age: 20.33 ± 2.43 y), participants entered an MRI scanner and performed the Think/No-Think (TNT) task wherein they attended to reminder cues (faces) that were each paired with an emotionally negative or neutral scene. For each reminder cue, participants either actively retrieved the corresponding scene (Think trials) or directly suppressed its retrieval (No-Think trials), and then indicated whether the reminder had evoked awareness of the scene (allowing us to isolate failed suppression attempts, referred to hereafter as intrusions). Experimental procedures are illustrated in Fig. 1.

#### Behavior.

**Fig. 2.**
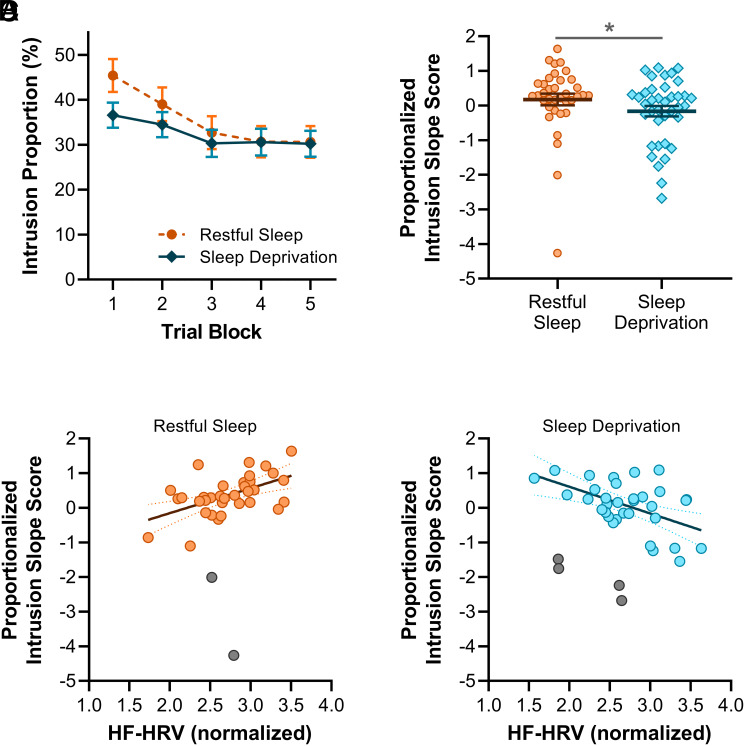
Adaptive memory suppression. (*A*) Intrusions decreased across trial blocks more rapidly after restful sleep as compared to sleep deprivation. (*B*) Intrusion slope scores were lower after sleep deprivation than restful sleep (higher intrusion slope scores indicate a greater reduction in intrusions over trials). (*C*) In the restful sleep group, HF-HRV was positively correlated with intrusion slope scores. (*D*) In the sleep deprivation group, HF-HRV was negatively correlated with intrusion slope scores. *P < 0.05.

We first assessed the impact of sleep deprivation on behavioral expressions of memory control. Repeatedly suppressing intrusive memories renders them less intrusive over time (2–5, 7, 8). Replicating this finding, participants showed overall decreasing numbers of intrusions (i.e., instances in which No-Think trials triggered awareness of the associated scene) across trial blocks (main effect: *F*(3.04,218.78) = 19.68, *P*< 0.001, *η_p_*^2^ = 0.21). This adaptive benefit of suppression was affected by group membership (trial block × group interaction: *F*(3.04,218.78) = 2.92, *P* = 0.035, *η_p_^2^* = 0.04; Fig. 2*A*), suggesting that sleep deprivation altered the rate at which suppressed memories became less intrusive over time. Indeed, sleep-deprived participants were significantly impaired in their ability to downregulate unwanted memories across TNT trial blocks relative to sleep-rested participants, as indicated by lower intrusion slope scores (*Materials and Methods*; *W* = 496, *P* = 0.043, *r_rb_* = 0.27; Fig. 2*B*). Adaptive suppression was unaffected by the emotional valence of the target scenes (trial block × valence interaction: *F*(4,288) = 0.60, *P* = 0.66, *η_p_^2^* < 0.01), and scene valence had no impact on the adaptive suppression deficit among sleep-deprived participants (trial block × group × valence interaction: *F*(4,288) = 1.34, *P* = 0.26, *η_p_^2^* = 0.02; *SI Appendix*, Table S1).

Although the intrusion slope results suggest that adaptive suppression improvement was impaired by sleep deprivation, there was no overall difference in intrusion proportion scores between sleep-deprived and sleep-rested participants (main effect: *F*(1,72) = 0.59, *P* = 0.44, *η_p_^2^* < 0.01), regardless of the emotional valence of the target scenes (group × valence interaction: *F*(1,72) = 0.04, *P* = 0.84, *η_p_^2^* < 0.01). This null effect, which contrasts our earlier work (10), may be partially explained by between-group differences in memory control ability at baseline. Indeed, in a mock TNT task on practice items that took place before the overnight interval (*Materials and Methods*), sleep-deprived participants reported fewer intrusions than sleep-rested participants, although the between-group difference across this limited number of trials did not reach statistical significance (sleep-rested group: M = 48.61%, SEM = 3.76%; sleep-deprived group: M = 38.82%, SEM = 3.42%; *F*(1,72) = 3.73, *P* = 0.058, *η_p_^2^* = 0.05). The same pattern emerged in the first block of the main TNT assessment phase (*t*(72) = 1.92, *P* = 0.059, *d* = 0.45; Fig. 2*A*), further supporting the notion that the sleep-deprived participants were naturally more effective memory suppressors than the sleep-rested participants. Notably, consistent with the finding that sleep-deprived participants showed a lower rate of improvement across trial blocks, the between-group difference in intrusion proportion scores was near zero by the final trial block (*t*(72) = 0.09, *P* = 0.93, *d* = 0.02). In an additional analysis that adjusted for individual differences in memory control ability at baseline (based on performance in the mock TNT task completed before the overnight delay), the overall pattern of results was more in keeping with our earlier work (10) in which sleep deprivation led to an overall increase in intrusion proportion scores (*SI Appendix*, Fig. S1). Consistent with previous studies (3, 10), intrusion proportion scores were not generally affected by the emotional valence of the target scenes (main effect: *F*(1,72) = 0.29, *P* = 0.59, *η_p_^2^* < 0.01).

#### HRV.

Inhibitory control over cognition is related to the high-frequency component (0.15 to 40 Hz) of heart rate variability [HF-HRV; (10, 12)], and this relationship may be modulated by sleep deprivation (10). To investigate how trait HF-HRV influenced the impact of sleep deprivation on mnemonic control, we recorded resting heart rate before the overnight interval. Interestingly, higher HF-HRV was associated with higher intrusion slope scores among participants who had slept (*r_skipped_* = 0.54 [0.22, 0.77]; Fig. 2*C*). This result suggests that, when well-rested, individuals with higher trait HF-HRV are more adept at suppressing unwanted memories. In contrast, among sleep-deprived individuals, higher resting HF-HRV was associated with lower intrusion slope scores (*r_skipped_* = −0.52 [−0.75, −0.22], Zou’s CI [0.66, 1.34]; Fig. 2). Further analysis of high-frequency and low-frequency components of resting HRV is available in *SI Appendix*, Fig. S2.

#### fMRI.

**Fig. 3.**
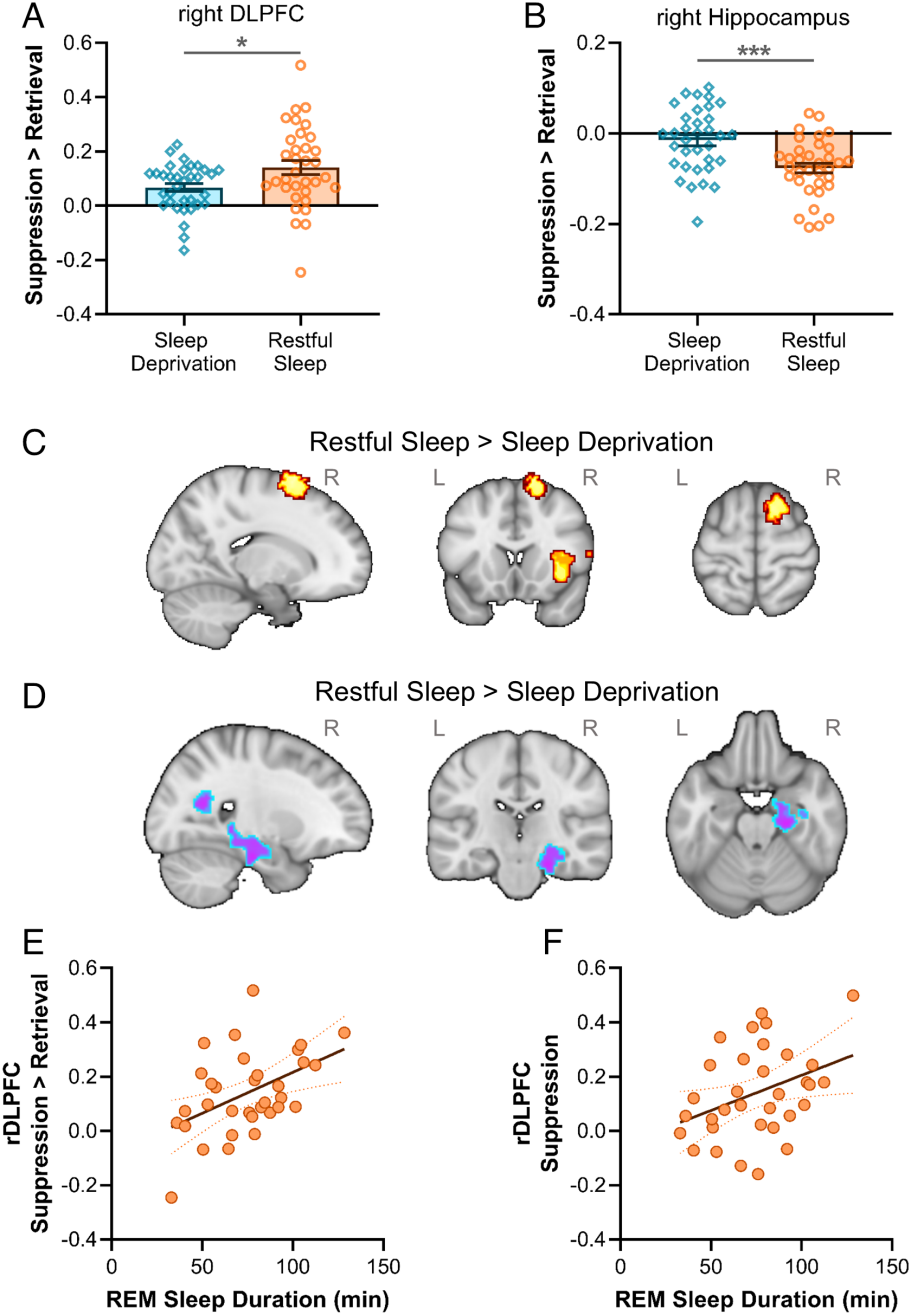
Functional brain responses during memory suppression. ROI analyses: (*A*) reduced engagement of the rDLPFC and (*B*) weaker disengagement of the right hippocampus after sleep deprivation relative to restful sleep, demonstrated by between-group differences in suppression > retrieval contrasts. Exploratory whole-brain analyses: (*C*) increased activation in the right superior frontal gyrus and right insular cortex, and (*D*) decreased activation in the right hippocampus after restful sleep (contrasted with sleep deprivation). (*E*) REM sleep duration was correlated with suppression-related activity in rDLPFC in the restful sleep group (suppression > retrieval contrast). (*F*) REM sleep duration was correlated with rDLPFC activation during suppression (as compared to baseline) in the restful sleep group, whereas no such effect was observed for activation during retrieval. *P < 0.05; ***P < 0.001, based on independent *t* tests. R = right hemisphere; L = left hemisphere.

We next examined the impact of sleep deprivation on the neural correlates of memory suppression. Downregulating memories in response to reminders has previously been shown to engage rDLPFC (3, 13–15, 39, 40). Replicating this finding, our rDLPFC region of interest (ROI), which was obtained from an independent meta-analytic conjunction analysis of domain-general inhibitory control (13), was more strongly engaged during memory suppression than retrieval (main effect: *F*(1,66) = 55.69, *P* < 0.001, *η_p_^2^* = 0.46). Critically, rDLPFC engagement during memory suppression was significantly lower after sleep deprivation than restful sleep (memory process × group interaction: *F*(1,66) = 5.68, *P* = 0.020, *η_p_^2^* = 0.08; Fig. 3*A*), suggesting that sleep deprivation impaired prefrontal memory control. Sleep deprivation (as compared to restful sleep) also led to a general reduction in rDLPFC activity (main effect: *F*(1,66) = 11.58, *P* = 0.001, *η_p_^2^* = 0.15). In keeping with prior work (3, 10), rDLPFC activity was not generally affected by scene valence (main effect: *F*(1,66) = 0.89, *P* = 0.349, *η_p_^2^* = 0.01), and scene valence did not modulate the effects of memory suppression or sleep deprivation on rDLPFC activity (all interactions involving valence: *P* ≥ 0.35).

Memory suppression orchestrated by rDLPFC has been shown to target retrieval operations in the hippocampus, especially in the right hemisphere (2, 6, 13, 14, 39, 40). In keeping with these prior findings, functional brain responses in our right hippocampus ROI were weaker during memory suppression than retrieval (main effect: *F*(1,66) = 29.42, *P* < 0.001, *η_p_^2^* = 0.31). Importantly, disengagement of the right hippocampus during memory suppression was diminished after sleep deprivation relative to restful sleep (memory process × group interaction: *F*(1,66) = 17.02, *P* < 0.001, *η_p_^2^* = 0.21; Fig. 3*B*), consistent with a failure to downregulate unwanted memories under conditions of sleep deprivation. No overall between-group difference was observed in the right hippocampus (main effect: *F*(1,66) = 3.12, *P* = 0.082, *η_p_^2^* = 0.05), there was no overall effect of scene valence (main effect: *F*(1,66) = 0.46, *P* = 0.50, *η_p_^2^* = 0.01), and the effects of memory suppression and sleep deprivation were not influenced by scene valence (all interactions involving valence: *P* ≥ 0.32).

We next investigated to what extent suppression-related activity in our ROIs (quantified hereafter as suppression > retrieval contrasts, unless stated otherwise) was associated with behavioral expressions of memory control. We found that greater suppression-related activity in rDLPFC was associated with lower intrusion slope scores, but only among sleep-rested individuals (*r_skipped_* = −0.40 [−0.63, −0.11]); no such correlation was observed in sleep-deprived individuals (*r_skipped_* = −0.21 [−0.51, 0.16], *Zou’s CI* [−0.62, 0.26]). Similarly, suppression-related rDLPFC activation was associated with higher intrusion proportion scores after restful sleep (*r_skipped_* = 0.42 [0.03, 0.69]) but not sleep deprivation (*r_skipped_* = 0.18 [−0.20, 0.46], *Zou’s CI* [−0.21, 0.67]). Earlier work has shown that rDLPFC activity increases when unwanted memories enter awareness and must be purged (2). Individuals in the restful sleep group who experienced more mnemonic intrusions may have thus required stronger prefrontal engagement to reactively suppress unsolicited retrieval operations than those who had fewer intrusions. This dynamic prefrontal control mechanism had presumably failed after sleep deprivation, preventing participants from reactively purging intrusive memory content. Suppression-related activity in the right hippocampus showed no relationship with intrusions in either sleep-deprived (intrusion slope: *r_skipped_* = −0.01 [−0.33, 0.33]; intrusion proportion: *r_skipped_* = 0.07 [−0.29, 0.35]) or sleep-rested individuals (intrusion slope: *r_skipped_* = −0.08 [−0.46, 0.31]; intrusion proportion: *r_skipped_* = 0.01 [−0.33, 0.34]).

An exploratory whole-brain analysis contrasting memory suppression and retrieval (collapsed across negative and neutral scenes) showed that suppression-related activity in the right superior frontal gyrus and right insular cortex was significantly higher in sleep-rested participants relative to sleep-deprived participants (Fig. 3*C*). Reciprocally, suppression-related activity in the right hippocampus was significantly lower after restful sleep than sleep deprivation (Fig. 3*D*). Together with the findings from our ROI analyses, these data are consistent with a breakdown of prefrontal memory control as a consequence of sleep deprivation. Such a breakdown could, theoretically, set the stage for hippocampal hyperactivity and increased vulnerability to intrusive memories. See Table 1 for the full set of results from our whole-brain analysis.

**Table 1.** fMRI whole-brain analysis

*X Y Z (MNI)*	*No. voxels*	*Activation*	*Region*	*Z-Max*
14 10 72	462	Increase	Superior frontal gyrus (right)	5.00
36 14 −4	338	Increase	Insular cortex (right)	3.96
14 −52 18	4688	Decrease	Precuneus (right)	4.67
4 22 −2	1752	Decrease	Subcallosal cortex (right)	4.26
22 −22 −22	649	Decrease	Parahippocampal gyrus (right)	3.98
−14 −86 2	350	Decrease	Intracalcarine cortex (left)	3.76

Activation changes associated with the contrast suppression > retrieval (collapsed across negative and neutral scenes) at the group level (restful sleep > sleep deprivation). X, Y, Z coordinates of the activation peak are shown in Montreal Neurological Institute (MNI) space. Cluster forming threshold *Z* = 3.1; *P* < 0.05 (FWE corrected).

### REM Sleep Restores Prefrontal Memory Control.

We next examined the role of REM sleep in the overnight restoration of memory control (see Table 2 for descriptive PSG data). Longer REM sleep duration was associated with greater suppression-related rDLPFC activity (*r_skipped_* = 0.47 [0.17, 0.71]; Fig. 3*E*). Confirming that this effect was specific to memory suppression, REM sleep duration was also significantly correlated with rDLPFC activity observed during suppression as compared to baseline (*r_skipped_* = 0.37 [0.07, 0.60]; Fig. 3*F*) but not retrieval as compared to baseline (*r_skipped_* = −0.06 [−0.40, 0.29]), and these correlation coefficients were significantly different (*Zou’s CI* [0.12, 0.71]). REM sleep duration was not significantly correlated with suppression-related activity in the right hippocampus (*r_skipped_* = 0.36 [−0.06, 0.71]). No significant correlation was observed between time spent in any non-REM sleep stage and suppression-related activity in rDLPFC (N1: *r_skipped_* = −0.21 [−0.47, 0.06]; N2: *r_skipped_* = 0.06 [−0.32, 0.45]; N3: *r_skipped_* = −0.02 [−0.36, 0.32]) or right hippocampus (N1: *r_skipped_* = −0.13 [−0.48, 0.22]; N2: *r_skipped_* = 0.21 [−0.21, 0.62]; N3: *r_skipped_* = 0.14 [−0.21, 0.45]). These findings indicate a role for REM sleep in restoring prefrontal control mechanisms underpinning the ability to prevent unwanted memories from entering conscious thought.

**Table 2.** Sleep PSG data

*N1*	*N2*	*N3*	*REM*	*TST*
23.45 (1.53)	226.78 (6.39)	76.10 (3.58)	69.50 (4.02)	395.83 (8.91)

Time (min) spent in each sleep stage and total sleep time (TST) in the restful sleep group. REM: rapid eye movement sleep; N1–N3: stages of non-REM sleep. Mean values are shown with SEM in parentheses.

### Sleep Deprivation Disrupts Adaptive Network Segregation.

**Fig. 4.**
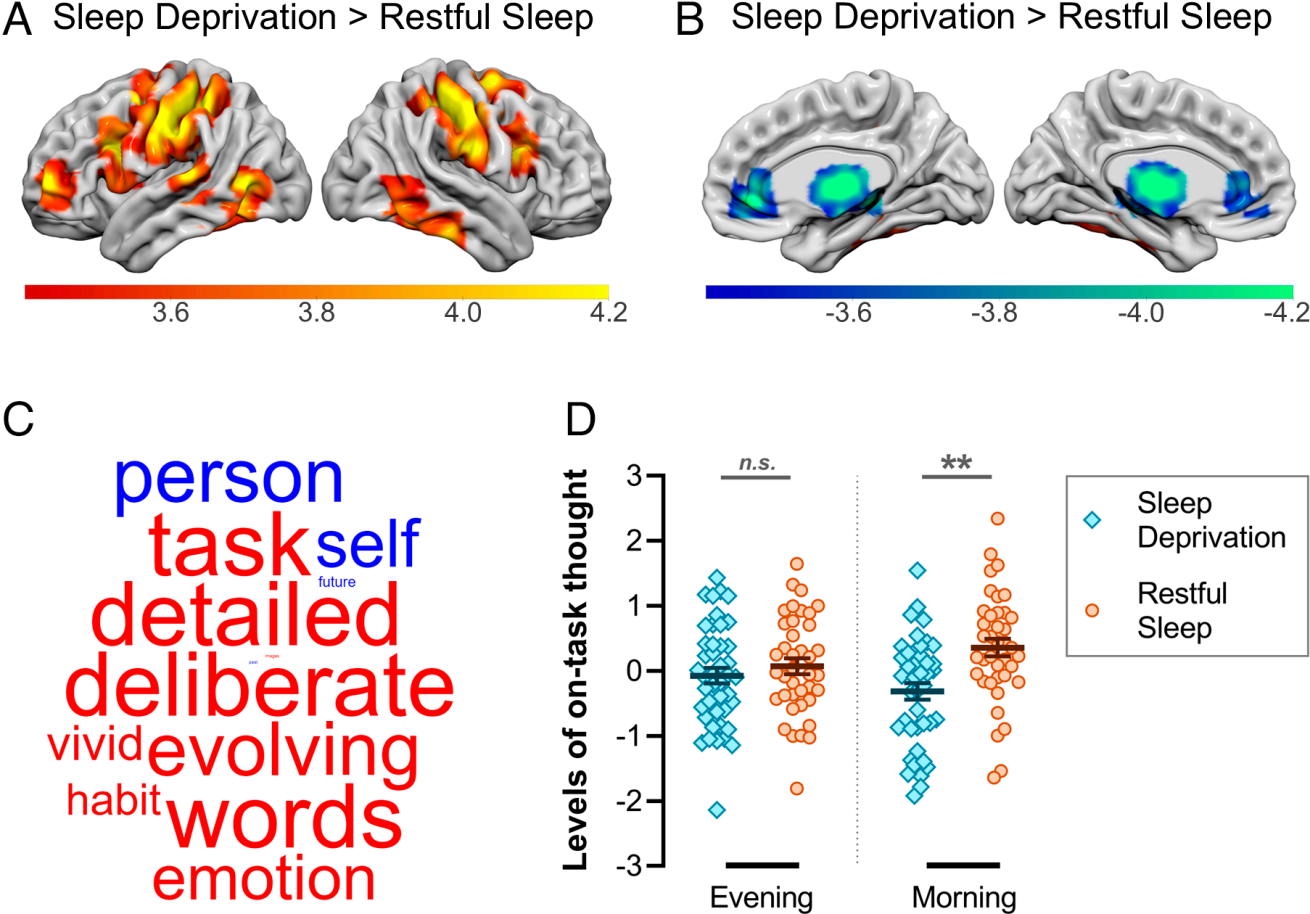
RS functional connectivity and self-generated patterns of thought. (*A*) Sleep deprivation (in contrast to restful sleep) increased functional connectivity between the DMN and several areas of the CCN. (*B*) Sleep deprivation (in contrast to restful sleep) decreased functional connectivity between the DMN and thalamus bilaterally. (*C*) The application of principal components analysis (PCA) to MDES data identifies latent patterns of thought by grouping thought probes that capture shared variance. The first component identified from the PCA corresponded to a pattern of *on*-task thinking. The loadings on this component are presented as a word cloud. The color of a word describes the direction of the relationship (red: positive, blue: negative) and the size of a word reflects the magnitude of the loading. (*D*) Patterns of on-task thinking were significantly reduced after sleep deprivation relative to restful sleep (no such difference emerged in the prior evening session). **P < 0.01; *n.s.* = nonsignificant.

So that we could assess the impacts of sleep deprivation on the brain’s intrinsic connectivity profile (i.e., in the absence of external task demands), participants underwent a RS fMRI scan after completing the TNT assessment phase. Sleep deprivation has been shown to disrupt functional decoupling between the DMN and CCN (23, 28), which support internally focused mental processing and externally directed cognitive control, respectively. In keeping with these earlier findings, DMN seed connectivity was significantly increased in a number of CCN areas—including bilateral middle frontal gyrus, inferior temporal gyrus, and supramarginal gyrus—after sleep deprivation relative to restful sleep (Fig. 4*A*). This suggests that sleep deprivation prevents the DMN from remaining functionally distinct from normally dissociable brain networks, giving rise to failures of cognitive control (see *SI Appendix*, Table S2 for the full set of clusters from our RS analyses).

Aberrant ascending input from the thalamus to cortical regions encompassing the DMN and CCN has been implicated in the collapse of brain network integrity after sleep deprivation (29). Consistent with this finding, sleep deprivation (as compared to restful sleep) significantly reduced DMN seed connectivity in the bilateral thalamus (Fig. 4*B*). However, for the CCN seed, no differences in thalamic connectivity were observed between the sleep-deprived and sleep-rested participants. Disruptions to thalamocortical connectivity arising from sleep deprivation thus appear to predominate in the DMN and, in doing so, may contribute to a breakdown of adaptive functional segregation between the DMN and brain networks underpinning externally focused cognition.

### Sleep Deprivation Reduces Deliberate Patterns of Thought.

Finally, we examined how sleep deprivation affects the content of ongoing thoughts under conditions of high and low cognitive demand. In the evening before sleep deprivation or restful sleep, and again in the morning, participants completed a task that varied in its requirements for external attention (Fig. 1*B*). The task alternated between a condition with minimal attentional demands (0 back) and a higher demand condition in which task-relevant information had to be maintained in working memory (1 back). At intermittent intervals, participants were asked to describe the contents of their ongoing thoughts using MDES—an established thought sampling technique that is sensitive to task contexts and daily activities (41–43). Specifically, they rated their recent experience along a series of thirteen thought probes, including whether their thoughts were focused on the task they were performing, were deliberate or spontaneous, or concerned past or future events (see *SI Appendix*, Table S3 for the full set of probes).

We applied PCA to the MDES data to identify latent patterns of thought (41–43). The first identified component captured an *on-task* dimension corresponding to deliberate and detailed thoughts about the task (Fig. 4*C*). Participants engaged in fewer of these on-task thoughts in the 0-back condition than in the relatively demanding 1-back condition (main effect: *F*(1,80) = 30.68, *P* < 0.001, *η_p_^2^* = 0.28), regardless of whether they were in the sleep-deprived or sleep-rested group (group × task interaction: *F*(1,80) = 0.52, *P* = 0.47, *η_p_^2^* < 0.01). Notably, while there was no group-level difference in on-task thinking at the evening session (before sleep deprivation or restful sleep, *t* = 0.82, *P* = 0.41), one did emerge the following morning, with sleep-deprived participants exhibiting less on-task thinking than those who had slept (*t* = 3.77, *P* = 0.001; group × session interaction: *F*(1,80) = 7.47, *P* = 0.008, *η_p_^2^* = 0.09; Fig. 4*D*). This effect was independent of task demands (group × task × session interaction: *F*(1,80) = 0.69, *P* = 0.41, *η_p_^2^* < 0.01). There was no overall difference in on-task thinking between the evening and morning sessions (main effect: *F*(1,80) = 0.05, *P* = 0.83, *η_p_^2^* < 0.01). These findings suggest that sleep deprivation impaired the ability to engage in deliberate, on-task thought, regardless of whether cognitive demands were high or low. See *SI Appendix*, Fig. S3 for analyses of the second component.

## Discussion

It is well established that sleep deprivation has a broad detrimental impact on higher-order cognition (25). Our findings demonstrate that these impairments extend to inhibitory memory control. Relative to sleep-rested participants, sleep-deprived participants were unable to properly engage rDLPFC during memory suppression, leading to a behavioral deficit in the ability to downregulate unwanted memories over time. Among sleep-rested participants, longer REM sleep duration was associated with greater suppression-related activity in rDLPFC, suggesting that REM sleep supports the overnight restoration of prefrontal memory control. Consistent with prior work (23, 28, 29), sleep deprivation led to aberrant patterns of functional connectivity between brain networks underpinning internally and externally focused cognition, as well as a tendency to engage in less deliberate, on-task thinking (30).

rDLPFC downregulates hippocampal retrieval operations in service of memory control (2, 3, 13, 14). We previously showed that sleep deprivation leads to a marked reduction in people’s ability to suppress unwanted memories (10), prompting our hypothesis that disruption to the prefrontal-hippocampal memory control network gives rise to memory suppression failures after prolonged wakefulness (16). Our findings provide robust support for this hypothesis: sleep deprivation not only reduced rDLPFC engagement during memory suppression, but also weakened suppression-related reductions in activity in the right hippocampus.

These findings add to a growing literature on the impacts of sleep deprivation on prefrontal control and underscore the importance of such findings for our understanding of mental health conditions that co-occur with chronic sleep disturbances. For example, sleep deprivation impairs prefrontal control mechanisms that resolve conflict between habitual and goal-directed behavior (44), providing mechanistic insight into the link between sleep disturbance and relapse in individuals with addictive disorders (45). Likewise, trait-anxious individuals and patients with anxiety disorders show reduced prefrontal activation and impaired functional coupling between the prefrontal cortex and amygdala during threat-related information processing (46, 47), a pattern that also emerges in healthy individuals deprived of sleep (20, 48). Given that memories play a central role in our affective perception of the external world (49), memory control failures may go a long way toward explaining the relationship between sleep deprivation and emotional dysregulation.

Along similar lines, we found that the magnitude of rDLPFC engagement during memory suppression was correlated with the amount of time that sleep-rested participants spent in REM sleep. Disturbances of REM sleep (e.g., reduced REM sleep latency) are commonplace in psychiatric disorders associated with intrusive and unwanted thoughts, including depression, anxiety, and posttraumatic stress disorder (33, 35, 37, 38). Furthermore, in healthy individuals, REM sleep has been implicated in affect regulation during exposure to emotionally aversive stimuli (31, 50). Taken together, these and the current findings raise the intriguing possibility that REM sleep supports the restoration of memory and emotional control processes mediated by the prefrontal cortex. Future work can address this possibility by directly manipulating REM sleep [e.g., via noninvasive auditory brain stimulation; (51)] and assessing its causal impacts on memory and affect suppression.

Our exploratory whole-brain analysis showed that sleep deprivation reduced activity in the right insular cortex during memory suppression. The right insular cortex plays a major role in switching between distinct brain networks across task paradigms and stimulus modalities (52), as is the case in our TNT protocol where participants must rapidly shift between conditions of memory retrieval (Think trials), and suppression (No-Think trials). Although speculative, this result might reflect an impact of sleep deprivation on the brain’s ability to switch between activation and deactivation of large-scale networks. In keeping with this idea, functional networks encompassing the right insular cortex show reduced activity when performing attention-demanding tasks after sleep deprivation (53). Relatedly, monitoring one’s own internal state (as is required when repeatedly switching between conditions with differing cognitive demands) may benefit from interoceptive processing, which recruits a network including the insular cortex (54), and is impaired by poor-quality sleep (55).

The findings from our RS fMRI data were consistent with prior evidence that sleep deprivation leads to aberrant patterns of functional connectivity between whole-brain functional networks supporting internally and externally focused cognition (23, 28). Specifically, we observed an increase in connectivity between the DMN and multiple areas of the CCN in sleep-deprived individuals, as compared to sleep-rested individuals. Moreover, DMN connectivity with bilateral thalamus was significantly reduced after sleep deprivation relative to restful sleep, as observed in earlier work (29). In the context of our TNT task, inappropriate gating of on-task relative to off-task network activity, together with a breakdown of ascending arousal input from the thalamus, is likely to impair adaptive switching between periods of rest (i.e., during fixation periods of up to 9,000 ms in duration) and trials that require active suppression of internally generated information (No-Think trials).

HF-HRV is linked to superior executive functioning, including memory and emotional control (10, 11). Consistent with this earlier work, our data revealed a significant correlation between HF-HRV at rest and memory suppression success over time. Importantly, whereas previous studies have linked HF-HRV to suppression-induced forgetting [i.e., lower recall for suppressed relative to baseline word pairs; (11)], this study demonstrates that resting cardiac activity is associated with a person’s ability to downregulate unwanted memories over time. This relationship was only observed in individuals who had obtained a night of restful sleep. For sleep-deprived participants, the opposite effect was observed: that is, higher resting HF-HRV was associated with lower memory suppression improvement across trials. Taken together, these results may suggest that people who are inherently resilient to intrusive memories (based on high resting HF-HRV) might also be more vulnerable to the effects of sleep deprivation on memory control—a possibility that should be addressed in future confirmatory research.

If sleep deprivation impairs the brain networks governing the suppression of unsolicited thoughts, then sleep-deprived individuals should show a reduction in deliberate and focused patterns of self-generated mental content relative to well-rested individuals. Our MDES protocol allowed us to address this question. Whereas the sleep-deprived and sleep-rested groups showed no difference in deliberate patterns of thought prior to the overnight delay (based on an on-task thought dimension derived from PCA of the MDES data), a group-level difference did emerge the following morning: sleep-deprived individuals reported fewer deliberate, on-task thoughts than sleep-rested participants, which is in keeping with prior work using self-reported categorizations of on-task and off-task thought (30). The effect of sleep deprivation on on-task thinking was unaffected by task demands, suggesting that sleep deprivation leads to changes in the content of endogenous thoughts that are impervious to external contexts.

Between-group differences in motivation cannot be ruled out as a contributory factor in our data. However, if sleep-deprived participants lacked motivation to complete the tasks as instructed, their performance would presumably have suffered in the n-back component of the MDES task that was administered at the end of the experiment (when sleep pressure was highest). No between-group difference in performance was observed on this measure (*SI Appendix*, Table S4), suggesting that differences in motivation are unlikely to account for the observed results.

Because caffeine is known to affect sleep and cognition (56), we asked all participants to abstain from caffeine during the overnight delay and for 24 h before the experiment (thereby preventing any between-group difference in caffeine consumption). It is possible that caffeine withdrawal among habitual caffeine consumers might have impacted the sleep manipulation and influenced task performance. Future work may circumvent this issue by including only nonhabitual caffeine consumers or by statistically adjusting for the possible influence of habitual caffeine use.

In conclusion, our findings demonstrate that sleep deprivation leads to widespread disruption of the brain networks supporting adaptive control. Sleep deprivation impaired rDLPFC engagement during memory suppression, increased suppression-related activity in the hippocampus, and disrupted RS connectivity between brain regions supporting internally and externally focused cognition (DMN and CCN, respectively). Among participants who obtained restful sleep, engagement of rDLPFC during memory suppression was correlated with time spent in REM sleep, suggesting that REM sleep might play a central role in the overnight restoration of prefrontal memory control mechanisms. The functional impairments arising from sleep deprivation were associated with a behavioral deficit in suppressing unwanted memories over time and coincided with a deterioration of deliberate, on-task thinking. Taken together, our findings highlight the critical role of sleep in maintaining control over memories and ongoing thoughts.

## Materials and Methods

### Participants.

Eighty-seven healthy adults aged 18 to 30 y completed the experiment. Participants were right-handed, native English speakers who declared that they typically awoke by 8 AM after at least 6 h of sleep and had no history of neurological, psychiatric, attention, or sleep disorders. Following standard procedures in our laboratory (57–61), participants were asked to abstain from alcohol and caffeine throughout the experiment and for 24 h prior. Written informed consent was obtained from all participants in line with the requirements of the Research Ethics Committee of the York Neuroimaging Centre at the University of York, who approved the study. Participants were compensated with £80 or experimental participation credit (University of York BSc Psychology students only).

Two participants were excluded from all analyses due to lack of appropriate engagement with the study protocol (e.g., persistently failing to follow instructions). The final sample included 85 participants, who completed either the sleep deprivation condition (n = 43; 18 males, mean ± SD age, 19.58 ± 1.72 y) or restful sleep condition (n = 42; 12 males, mean ± SD age, 20.33 ± 2.43 y). On the night before the experiment, 1 participant (1.18%) reported obtaining less than 6 h of sleep (5 h 45 min) and 10 participants (11.76%) reported getting up after 8 AM.

### Procedure.

Two sessions (evening and morning) were separated by a night of sleep deprivation or restful sleep (Fig. 1*A*). Participants collected an actigraphy wristwatch (Philips Respironics, Murrysville, PA) at 9 AM on the day of the evening session so we could ensure they had not napped during the day (confirmed via subsequent analysis of actigraphy data). Hence, by the time of the morning session, participants in the sleep deprivation group had been awake for ~24 h.

#### Evening.

T1 structural MRI scans were collected in the evening (~6 PM) to ensure that participants were comfortable in the MRI environment before committing to the full experiment. After participants exited the scanner, an 8-min resting ECG recording was obtained for the purpose of calculating heart rate variability (HRV). Participants were instructed to sit with their hands on their lap, relax, and breathe normally throughout.

We then administered the MDES task (Fig. 1*B*). Experience was sampled in a task that included nontarget and target trials and switched between conditions of 0-back and 1-back for the purpose of manipulating working memory demands. Nontarget trials were identical in the 0-back and 1-back conditions and consisted of two different shapes (circles, squares, or triangles) separated by a center line (jittered duration from 500 to 1,500 ms). The color of the center line indicated to the participant which condition they were in (blue = 0-back; red = 1-back). Nontarget trials did not require a behavioral response from participants and were presented in runs of 2 to 5 trials, after which a target trial or MDES probe was presented. On target trials, participants were required to indicate the location of a particular shape (circle, square, or triangle; jittered duration from 3,500 to 5,000 ms). The cognitive demand required to fulfill this instruction depended on whether participants were in the 0-back or 1-back condition. In the 0-back (nondemanding) condition, for target trials, two different shapes were presented on either side of the center line (as in the nontarget trials), and an additional shape appeared in the middle of the center line. Participants were required to indicate (via button press) which of the lateral shapes matched the central shape. Hence, in the 0-back condition, nontarget trials did not require continuous monitoring and participants could make perceptually guided decisions. In the 1-back condition, target trials also involved a shape appearing in the middle of the center line, but with a question mark on either side (instead of shapes). Participants were required to indicate which of the two lateral shapes from the previous trial matched the present central shape. Target trial decisions in the 1-back condition were therefore guided by working memory, meaning that the participants were required to continuously monitor the nontarget trials. All participants completed the 0-back and 1-back conditions once (order counterbalanced), with two target trials per condition. Patterns of ongoing thought were measured using MDES probes, which occurred instead of target trials on a quasi-random basis. For the MDES probes, participants were asked to what extent their thoughts were focused on the task, followed by 12 randomly shuffled questions about the content of their thoughts (*SI Appendix*, Table S3). The questions were administered four times (twice in each of the 0-back and 1-back conditions) and participants made their responses on a sliding scale ranging from 1 to 10.

Affect ratings were then collected for 68 scenes. Each scene was presented for 6,500 ms and participants were instructed to focus their attention on the image for the entire time. They were then shown a 9-point pictorial rating scale that ranged from a frowning face on the far left (depicting feelings of extreme sadness or displeasure) to a smiling face on the far right (depicting feelings of extreme happiness or pleasure). Participants were given 15,000 ms to provide their affect rating (1 to 9) via button press, but were asked to respond quickly and spontaneously. Trials terminated after an affect rating had been provided or the 15,000 ms time limit expired, after which a fixation cross appeared for 500, 1,000, 1,500, or 2,000 ms. Affect rating data are available in *SI Appendix*, Table S5.

Participants then completed the TNT learning phase, which involved encoding pairwise associations between faces and scenes (Fig. 1*C*). On each trial, participants were shown a face and scene together for 6,000 ms and instructed to form a rich connection between them. Faces were always emotionally neutral whereas half the scenes were negative and the other half were neutral. Participants then completed a reinforcement phase where they were shown each of the faces in isolation for up to 4,000 ms and indicated via button press whether or not they could visualize the corresponding scene. When participants indicated that they could visualize the scene, they were shown the correct scene alongside two additional scenes from the learning phase that were not paired with the face and asked to select the correct image. If participants selected the correct scene, the face-scene pair was dropped from the reinforcement phase. If they failed to select the correct scene within 5,000 ms or they had indicated that they could not visualize the scene associated with the face, the face-scene pair was retained in the reinforcement phase, and they were tested on it again later in the same phase. Regardless of their response, participants were always shown the correct face-scene pairing for 3,500 ms at the end of the trial to reinforce their knowledge of the pairs. The reinforcement phase continued until participants had correctly identified the target image associated with each face cue. Participants then completed the entire reinforcement phase again. This “overtraining” procedure was used to ensure that participants would find it difficult to prevent the scenes from automatically intruding into consciousness when presented with the face cues during the TNT assessment phase. Forty-eight face-scene pairs (24 negative, 24 neutral) were presented at the learning phase and used in the main TNT assessment. Twelve additional face-scene pairs (6 negative, 6 neutral) served as fillers that were also used in the mock TNT assessment.

The mock version of the TNT assessment phase was then administered (outside of the MRI scanner) to enable participants to practice engaging in memory suppression. On each trial, a face cue was presented in isolation inside a green frame (*Think* trial) or red frame (*No-Think* trial). For green-framed faces, participants were instructed to visualize—in as much detail as possible—the scene associated with the face for the entire 3,000 ms it was presented. For red-framed faces, participants were instructed to focus their attention on the face for the entire 3,000 ms, but simultaneously prevent the associated scene from coming to mind. Participants were instructed to accomplish this by making their mind go blank, rather than by generating diversionary thoughts such as alternative images, thoughts, or ideas (3, 8, 10). If the scene came to mind automatically, participants were asked to actively push the scene out of mind. At the offset of each face cue, participants reported whether the scene paired with the preceding face had entered conscious awareness during the trial by pressing (with their right hand) a button corresponding to one of three options: *never*, *briefly*, or *often*. Participants were instructed to provide a response of *never* if the scene never entered awareness at all during the trial. Conversely, participants were instructed to respond with *briefly* if the scene briefly entered conscious awareness at any time during the trial, or with *often* if the scene entered awareness several times or for a period longer than what one would consider “brief.” These intrusion ratings were collected to determine how competent participants were at suppressing scenes associated with faces for No-Think trials. Although participants had up to 4,000 ms to make this rating, they were instructed to respond quickly, without dwelling on their decision. Participants moved immediately onto the next trial after providing their rating (jittered fixation of 500, 2,000, 2,500, 4,000, or 6,500 ms). The mock TNT assessment included only faces from the 12 face-scene pairs used as fillers at learning, and these were equally divided between conditions (6 Think, 6 No-Think; each 3 negative, 3 neutral).

#### Overnight.

The restful sleep group were fitted with electrodes for sleep PSG. Lights were turned out at approximately 10 PM and participants were awoken at 6 AM, providing an 8 h sleep opportunity. The 6 AM wake-up time was necessary to ensure the restful sleep group completed the TNT assessment at approximately the same time as the sleep deprivation group (accounting for the time taken to remove PSG electrodes, etc.). The sleep deprivation group remained awake across the entire night either in the lab under the supervision of an experimenter (n = 23) or at home (n = 20). Some participants stayed awake in the lab and others at home due to UK Government social distancing guidelines during the SARS-CoV-2 pandemic, which were enforced part-way through the experiment. To ensure that participants who stayed awake at home adhered to the sleep deprivation protocol, they were asked to send an SMS message to the experimenter every 30 min throughout the night. This requirement was fulfilled by all 20 participants. Wristwatch actigraphy also confirmed that these participants remained awake throughout the night. Whether the sleep deprivation group spent the overnight delay in the lab or at home had no significant influence on our main measures of interest (*SI Appendix*, Fig. S4). During the overnight phase, the sleep-deprived participants were permitted to read, use personal computers or other devices, watch TV, or play games. They were also permitted to eat and drink at any time, but caffeine was prohibited.

#### Morning.

A second ECG recording was obtained from each participant under the same conditions as the preceding session. Participants then entered the MRI scanner. We first administered a memory refresher phase where each of the face-scene pairs were presented for 1.5 s, allowing participants to reinforce their knowledge of the pairs (no scanning was performed at this time). The TNT assessment phase proper was then administered in 5 blocks while participants were undergoing fMRI. This followed the same procedures as the mock TNT assessment, but included faces from the main 48 face-scene pairs presented at learning (faces from the filler pairs used in the mock TNT assessment were not included here) and had a jittered fixation of 500 to 9,000 ms between trials. Each block lasted approximately 8 min and included two repetitions of 16 Think (8 negative, 8 neutral) and 16 No-Think (8 negative, 8 neutral) items presented in pseudorandom order (with the two repetitions of each item appearing at least three trials away from one another). Participants therefore completed a total of 320 trials (32 trials × 2 conditions × 5 blocks). The remaining 16 scenes that were included in the TNT learning phase did not appear in the TNT assessment phase. These baseline images were used in the affect rating task to index generalized changes in emotional reactivity that could be compared to changes arising from mnemonic suppression (*SI Appendix*, Table S5). A B0 fieldmap was acquired between the second and third blocks of the TNT assessment.

Participants then underwent a 9-min RS fMRI scan and were instructed to focus on a fixation cross in the center of the screen throughout. After participants exited the scanner, we collected another round of affect ratings and administered the MDES paradigm again, following identical procedures to the evening session. Finally, participants completed a postexperiment questionnaire, which probed how closely they had followed the TNT task instructions (62). The postexperiment questionnaire is available as *SI Appendix*, Survey S1.

### Stimuli.

Forty-eight emotionally neutral face images (24 male, 24 female) served as cues in the TNT task, and an equal number of scene images [24 negative, 24 neutral; (63)] served as targets. Face-scene pairs were created by randomly assigning each face cue to a target scene. Three lists of 16 pairs (8 negative; 8 neutral) were created from the 48 pairs to allow three within-subjects TNT conditions (Think, No-Think, and Baseline). A second version of each of the three lists was also created by assigning a new face cue to each target scene within a given list. The assignment of face-scene pairs to TNT conditions was counterbalanced across participants, as was the version of face-scene pairings within the TNT condition. Twelve additional face-scene pairs (6 negative, 6 neutral) were created for use as fillers. The same 60 scenes (48 experimental + 12 fillers) were used for the affect rating task, which featured a further 8 filler scenes (4 negative, 4 neutral).

### Software.

The TNT and MDES tasks were run in Presentation version 20.3 (Neurobehavioral Systems, Albany, CA) and PsychoPy2 (64), respectively. Tasks administered outside of the MRI scanner were run on a desktop PC with flat-screen monitor or laptop, and responses were collected using the keyboard.

### MRI Data Acquisition.

MRI scans were performed at York Neuroimaging Centre, University of York, UK, with a Siemens MAGNETOM Prisma 3T scanner. All scans were acquired with a 64-channel head coil, with whole brain coverage.

#### T1 Structural Scan.

A high-resolution T1-weighted structural scan was acquired with a 3D magnetization-prepared rapid gradient-echo (MPRAGE) sequence (TR = 2,300 ms, TE = 2.26 ms, flip angle = 8°, field of view = 256 mm, isotropic voxel size = 1 mm, 176 slices, slice thickness = 1 mm, anterior-to-posterior phase encoding direction, in-plane acceleration factor = 2 [GRAPPA]).

#### fMRI.

Event-related changes in blood oxygen level-dependent (BOLD) signal were acquired with a T2*-weighted gradient echo-planar imaging (EPI) sequence (TR = 2,000 ms, TE = 30 ms, flip angle = 75°, field of view = 240 mm, isotropic voxel size = 3 mm, 70 slices, slice thickness = 3 mm, interleaved slice acquisition, anterior-to-posterior phase encoding direction, in-plane acceleration factor = 2 [GRAPPA]). Five event-related fMRI scans were acquired per participant (corresponding to 5 blocks of the TNT assessment phase; referred to hereafter as TNT scans). Each scan was preceded by dummy volumes to allow for steady-state magnetizations to become established (at which time the scanner’s trigger signal initiated the task in synchrony with the acquisition of the first fMRI volume proper). Stimuli were projected onto a screen at the rear of the magnet bore with a PROPixx DLP LED Projector (VPixx Technologies, Inc.) and comfortably viewed with an angled mirror attached to the head coil. Participant responses were captured with a USB button response box placed in their right hand.

A 9-min RS fMRI scan was acquired immediately after participants completed the TNT task with a gradient EPI sequence (TR = 2,000 ms, TE = 30 ms, flip angle = 80°, field of view = 240 mm, isotropic voxel size = 3 mm, 70 slices, slice thickness = 3 mm, interleaved slice acquisition, anterior-to-posterior phase encoding direction, in-plane acceleration factor=2 [GRAPPA]).

#### B0 Fieldmap.

We acquired a B0 fieldmap to correct for magnetic field inhomogeneities in the fMRI data (TR = 850 ms, TE1 = 4.92 ms, TE2 = 7.38 ms, flip angle = 60°, field of view = 240 mm, isotropic voxel size = 3 mm, 72 slices, slice thickness = 3 mm, interleaved slice acquisition, right-to-left phase encoding direction). The B0 fieldmap was acquired between the second and third TNT block. If participants exited the scanner for a break between scans (sleep-rested group: n = 2, sleep-deprived group: n = 1), a second fieldmap was acquired after they reentered the scanner. This second B0 Fieldmap was used to correct for magnetic field inhomogeneities in the remaining scans.

### HRV.

ECG was recorded using a BIOPAC MP36R data acquisition system and AcqKnowledge version 4.4.1. Three BIOPAC EL503 ECG electrodes were attached to the midline of the left and right clavicle and the lower left rib. ECG was recorded continuously at a sampling rate of 2 KHz for 8 min.

### PSG.

Sleep was recorded using an Embla N7000 PSG system (Embla Systems, Broomfield, CO) and RemLogic version 3.4. The scalp was cleaned with NuPrep exfoliating agent, before gold-plated electrodes were attached using SAC2 electrode cream. Electroencephalography (EEG) electrodes were attached at eight standard locations according to the international 10–20 system (Homan, Herman, & Purdy, 1987): F3, F4, C3, C4, P3, P4, O1, and O2, each referenced to the contralateral mastoid (A1 or A2). Left and right electrooculogram, left, right, and upper electromyogram, and a ground electrode (forehead) were also attached. All electrodes were verified to have a connection impedance of < 5 kΩ and were sampled at a rate of 200 Hz.

To calculate the time spent in each stage of sleep, the PSG recordings were partitioned into 30 s epochs and scored, using RemLogic software, as wakefulness, N1, N2, N3, or REM sleep in accordance with the criteria of the American Academy of Sleep Medicine (65).

### Data Analysis.

Data were analyzed using JASP 0.13.1.0 (JASP Team, Amsterdam, The Netherlands), unless specified otherwise.

#### Behavior.

Although intrusion reports for each trial of the TNT assessment phase were obtained on a 3-point scale (*never*, *briefly*, *often*), participants rarely gave ratings of *often* for No-Think trials (mean ± SEM, 2.47 ± 0.49 %). For simplicity, we therefore followed our prior work (10) by combining the *briefly* and *often* responses, rending the judgment binary. Intrusion proportion scores were robustly greater for Think trials (mean ± SEM, 93.25 ± 0.92 %) as compared to No-Think trials (mean ± SEM, 34.01% ± 2.11 %; *t*(73) = 25.22, *P* < 0.001, *d* = 2.93), demonstrating that participants were generally effective at voluntarily preventing unwanted memories from entering awareness. Because our research questions concern involuntary memory intrusions, our analyses focus exclusively on No-Think trials.

Intrusion proportion scores were applied to a mixed measures ANOVA with factors Group (Sleep Deprivation, Restful Sleep), Emotion (Negative, Neutral), and Trial Block (1, 2, 3, 4, 5). Mauchly’s test of sphericity indicated that, for some effects, the assumption of sphericity was violated. In these cases, Greenhouse–Geisser correction was applied. To directly quantify adaptive memory suppression (i.e., the rate at which intrusions decreased over time), we computed an intrusion slope score for each participant. Intrusion slope scores were calculated by taking the slope of the linear regression line through intrusion proportion scores (averaged across scene valence categories) across TNT trial blocks. This value was divided by the participant’s intrusion proportion score in the first block to account for the fact that initial intrusion rates varied and participants with more initial intrusions had a greater margin for reducing their intrusion frequency. Accordingly, intrusion slope scores were not significantly correlated with intrusion proportion scores: *r_skipped_* = −0.13 [−0.44, 0.12]. We then multiplied the values by −1 to render the (primarily) negative scores positive. Increasing positive scores thus reflect increasing levels of competency at downregulating the frequency of intrusions over time. This measure was z normalized within the participant’s stimuli counterbalancing group (4, 5, 10), which allowed us to quantify intrusion slope scores with respect to a group of participants who attempted to suppress/retrieve precisely the same scenes in the TNT task. Shapiro–Wilk tests indicated that intrusion slope scores were not normally distributed in either group (both *P* < 0.05), so nonparametric Mann–Whitney U tests were used to compare intrusion slope scores between the sleep-deprived and sleep-rested groups. N = 11 participants were excluded from the TNT behavioral analyses due to: revealing in the postexperiment questionnaire they had engaged in unsolicited behaviors during the TNT task [e.g., intentionally thinking about scenes associated with red-framed faces, quantified as a score ≥5 on Question 2 of the postexperiment questionnaire available as *SI Appendix*, Survey S1; (62)], indicating that they had not understood the task instructions (n = 2), or data loss due to a technical fault (n = 1).

Consistent with earlier studies investigating the neural mechanisms of memory control with the TNT task (3, 15, 66), skipped Pearson’s correlations [MATLAB toolbox: Robust Correlation; (67)] were used to investigate linear relationships between variables (e.g., functional brain activity and time spent in REM sleep). Skipped correlations detect and ignore outliers by considering the overall structure of the data, providing accurate false positive control without loss of power. We compared the skipped correlations between groups using Zou’s CI [R package: cocor; (68)]. Skipped correlations were interpreted as significantly different if Zou’s CI did not contain zero (68). N = 2 participants were excluded from correlational analyses involving PSG data due to EEG electrodes becoming detached during the night (preventing accurate calculation of time spent in each sleep stage).

#### MDES.

PCA was carried out in SPSS version 27.0.1.0. The scores from the 13 experience sampling questions were entered into a PCA to describe the underlying structure of the participants’ responses. Because the 13 thought probes were administered twice during each of the 0-back and 1-back blocks, the two responses to each probe were averaged across iterations. Following our prior work (43), we concatenated the 13 responses of each participant in each session (evening/morning) and task (0-back/1-back) into a single matrix and carried out a PCA with varimax rotation. Two components were selected based on the inflection point in the scree plot (*SI Appendix*, Fig. S3), cumulatively explaining 42.77% of the total variance. The first component corresponded to a pattern of on-task thought, based on its question loadings. Scores for the two components were extracted for each participant and entered into a mixed-measures ANOVA with factors Task (0-back, 1-back), Session (Evening, Morning), and Group (Sleep Deprivation, Restful Sleep). Significant interactions were interrogated using Holm–Bonferroni post hoc comparisons. N = 3 participants were excluded from the MDES analysis due to their data not being acquired in both sessions (N = 2) or providing very high ratings to an unrealistic number of thought probes (N = 1; mean response of 9.3/10, which was >4 SD above the group mean).

#### fMRI.

Event-related fMRI data were analyzed using the FMRIB Software Library (FSL version 5.0; https://fsl.fmrib.ox.ac.uk/fsl/fslwiki/FEAT/). T1-weighted structural brain images were extracted using the Brain Extraction Tool (BET) and the functional data were preprocessed and analyzed using the fMRI Expert Analysis Tool (FEAT). Individual TNT scans were subjected to motion correction using a six-parameter rigid body transformation, B0 unwarping, and slice-timing correction. TNT scans were coregistered with the relevant structural image using Boundary-Based Registration (69) and were spatially normalized to the Montreal Neurological Institute (MNI-152) canonical brain. Functional images were spatially smoothed using a Gaussian kernel of FWHM 8 mm and underwent high-pass temporal filtering (Gaussian-weighted, 100 s).

A general linear model [GLM; (70)] was set up for each participant and TNT scan. This included four explanatory variables (EVs) to model retrieval of negative scenes (EV1: Think/Negative), retrieval of neutral scenes (EV2: Think/Neutral), suppression of negative scenes (EV3: No-Think/Negative) and suppression of neutral scenes (EV4: No-Think/Neutral). All the EVs were convolved with a canonical hemodynamic response function, and their temporal derivatives were also added to the model. Six movement parameters, acquired during motion correction, were included as nonconvolved nuisance regressors. Contrast images were obtained for each effect of interest (Think/Negative, Think/Neutral, No-Think/Negative, No-Think/Neutral). For each participant, the parameter images corresponding to each TNT scan were taken forward to a higher-level, fixed-effects model to average across activity within individuals.

Our ROI analyses focused on rDLPFC and right hippocampus, as these regions have been heavily implicated in memory suppression. For the rDLPFC ROI, we constructed a 5 mm sphere centered at MNI coordinates 35, 45, 24, which were obtained from an independent meta-analytic conjunction analysis of domain-general inhibitory control (13). The ROI for the right hippocampus was taken from the Harvard-Oxford subcortical atlas (http://fsl.fmrib.ox.ac.uk/fsl/fslwiki/Atlases) and was restricted to voxels with ≥50% probability of belonging to the right hippocampus. Individual parameter estimates were extracted from the higher-level fixed-effects analyses (averaging across TNT scans in each participant) and averaged in each ROI (using the featquery tool). The ROIs were then separately analyzed using a mixed-measures ANOVA with factors Group (Sleep Deprivation, Restful Sleep), Memory Process (Retrieval, Suppression), and Emotion (Negative, Neutral). Given no effects of Emotion emerged in either ROI, we collapsed across valence categories in subsequent Suppression > Retrieval contrast analyses.

A complementary whole-brain analysis was conducted in FSL to permit further investigation of how sleep deprivation affects the neural correlates of memory suppression. Because our ROI analyses revealed no significant effects of Emotion, we set up new GLMs for each participant and TNT scan with two EVs (Think and No-Think) that were both collapsed across negative and neutral scenes (the set-up for this first-level analysis was otherwise identical to that reported above). A contrast was obtained for the effect of No-Think > Think (i.e., suppression > retrieval), and the resulting contrast images for each TNT scan were averaged within participants using a higher-level fixed-effects analysis. Participant contrast images were then used as input for the group-level (mixed-effects) analysis using FLAME, as implemented in FSL, with a cluster forming threshold of *Z* = 3.1 and *P* < 0.05 family-wise error correction. N = 17 participants were excluded from the event-related fMRI analyses due to: being excluded from the behavioral analysis (n = 11; see *Data Analysis* for further details), poor coregistration between the EPI and structural images (n = 2), or loss of ≥3 (out of 5) TNT scans due to excessive movement (relative displacement >3 mm) and/or major artifact in the EPI data (n = 4). For participants with excessive movement detected on ≤2 TNT runs (relative displacement >3 mm), those runs were excluded and higher-level analyses were conducted on the remaining data (only 7 of 340 runs were excluded in total; sleep-rested group: 2 runs, sleep-deprived group: 5 runs).

RS fMRI data were analyzed using the CONN functional connectivity toolbox version 21.a [https://web.conn-toolbox.org/; (71)], implemented with SPM version 12 (https://www.fil.ion.ucl.ac.uk/spm/) and MATLAB version 2019a. Preprocessing steps followed CONN’s default pipeline, which included motion correction using a six-parameter rigid body transformation, slice-time correction, and the simultaneous segmentation and normalization of gray matter, white matter, and cerebrospinal fluid within both the fMRI and T1-weighted structural data to the MNI-152 canonical brain. Next, we removed potential confounding effects from the BOLD signal using linear regression, including the six movement parameters acquired during motion correction and their 1st and 2nd order derivatives, volumes with excessive movement (motion greater than 0.5 mm and global signal changes larger than z = 3), signal linear trend, and five principal components of the signal from white matter and cerebrospinal fluid [CompCor; (72)]. A bandpass filter of 0.01 to 0.1 Hz was also applied to the data.

Seed-based connectivity maps were calculated for each participant with the DMN and frontoparietal CCN as ROIs [as derived from the Yeo 7-network parcellation; (73)]. Nuisance regressors included movement parameters calculated during motion correction, volumes with excessive movement and framewise displacement. The participant connectivity maps were then used as input for the group-level analysis, which included age, gender, and mean framewise displacement as mean-centered covariates. All connectivity results were thresholded using Gaussian random field theory, as implemented in CONN, with a *P* < 0.001 (uncorrected, two-sided) voxel threshold and an FDR-corrected cluster threshold of *P* < 0.05/7 = 0.007, to also correct for testing multiple seeds (Bonferroni correction). N = 6 participants were excluded from the RS analysis due to: poor coregistration between the EPI and structural images (n = 2), excessive movement (n = 2), a major artifact in the structural image (n = 1), or a neurological anomaly (n = 1; note that this participant was also excluded from the behavioral and event-related fMRI analyses for indicating that they had misunderstood the TNT task instructions).

#### HRV.

The first 2-min and last 1-min of each 8-min ECG recording were excluded and HRV was calculated using the remaining 5-min of data. R-peaks were automatically detected using AcqKnowledge’s QRS detection algorithm before being visually inspected for accuracy. Peaks that the algorithm missed or erroneously detected were manually inserted or deleted, respectively. The interbeat-interval time series was then imported to Kubios version 3.0.2. for analysis. Autoregressive estimates of low-frequency (0.04 to 0.15 ms^2^/Hz) and high-frequency (0.15 to 0.40 ms^2^/Hz) power were used to obtain frequency-domain-specific indices of HRV. In keeping with earlier work (12, 74), values of low-frequency HRV (LF-HRV) and high-frequency HRV (HF-HRV) were transformed logarithmically (base 10). N = 1 participant was excluded from all HRV analyses because the ECG revealed signs of cardiac arrhythmia.

## Supplementary Material

Appendix 01 (PDF)

## Data Availability

Study data is publicly available via the following link: https://osf.io/jfdbx/?view_only=175ffc1f9e9249e09d27612cfcaf62ec (75).
