# Supplementary material for: Memory control deficits in the sleep-deprived human brain
Source: Proc Natl Acad Sci U S A. 2024 Dec 31;122(1):e2400743122. doi: 10.1073/pnas.2400743122 (PMC11725914; doi:10.1073/pnas.2400743122)
Supplement: Supplementary file 1 — Appendix 01 (PDF) [file pnas.2400743122.sapp.pdf]

PNAS

Supporting Information for
Memory control deficits in the sleep-deprived human brain

Marcus O. Harrington, Theodoros Karapanagiotidis, Lauryn Phillips, Jonathan Smallwood, Michael C. Anderson, and Scott A. Cairney*

* Correspondence: scott.cairney@york.ac.uk

This PDF file includes:

Figures S1 to S4
Tables S1 to S5
Survey S1

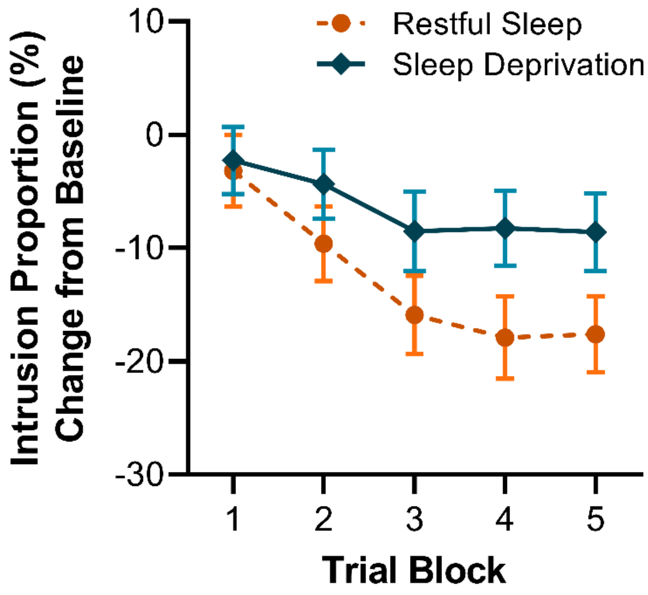

Supplementary Figure 1. Intrusion proportion scores adjusted for individual differences at baseline. To account for between-group differences in memory control ability, we subtracted intrusion proportion scores obtained during the evening mock Think/No-Think (TNT) task from intrusion proportion scores obtained during each block of the morning TNT assessment phase. These baseline-adjusted scores were then submitted to a mixed 2 (Group: Sleep Deprivation, Restful Sleep) x 5 (Trial Block: 1, 2, 3, 4, 5) ANOVA. There was a significant reduction in intrusions across trial blocks (main effect: $F(3.06, 220.61)=19.18$, $p<.001$, $\eta_p^2=0.21$, *Greenhouse-Geisser corrected*), and this reduction was modulated by group membership (trial block x group interaction: $F(3.06, 220.61)=2.77$, $p=.041$, $\eta_p^2=0.04$, *Greenhouse-Geisser corrected*). The main effect of group was non-significant ($F(1, 72)=2.25$, $p=.138$, $\eta_p^2=0.03$).

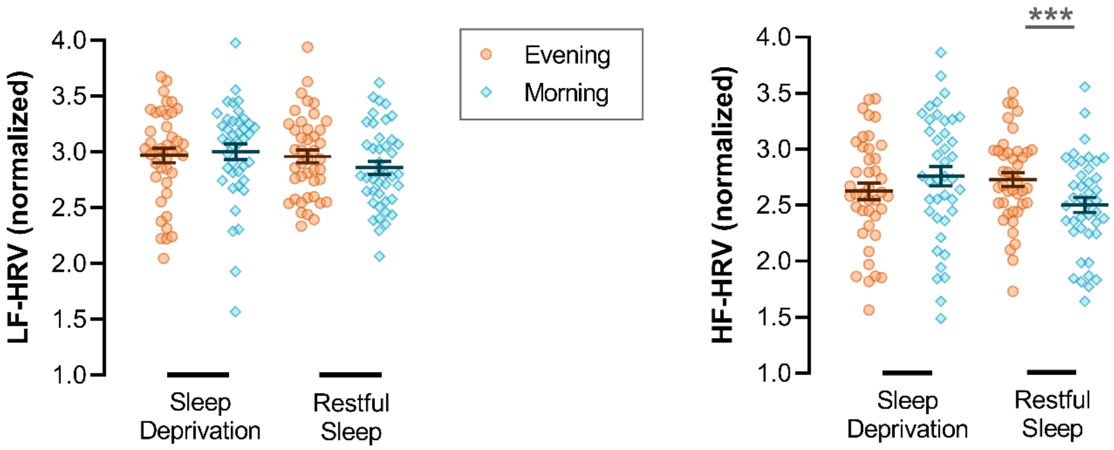

Supplementary Figure 2. Low-frequency and high-frequency heart rate variability (LF-HRV; HF-HRV). To investigate the impact of sleep deprivation on HRV, our indices of HF- and LF-HRV were applied to mixed 2 (Group: Sleep Deprivation, Restful Sleep) x 2 (Session: Evening, Morning) ANOVAs. Post-hoc comparisons were conducted with Holm-Bonferroni correction. LF-HRV (left panel) did not differ significantly between the evening and morning sessions (F(1,81)=0.95, p=.33, η_p^2=0.01) and was not influenced by sleep deprivation (as compared to restful sleep) in either session (main effect: F(1,81)=0.89, p=.35, η_p^2=0.01; interaction: F(1,81)=3.54, p=.063, η_p^2=0.04). HF-HRV (right panel) was also comparable between the evening and morning sessions (F(1,81)=1.39, p=.24, η_p^2=0.02) and there was no general effect of sleep deprivation (as compared to restful sleep; F(1,81)=0.63, p=.43, η_p^2<0.01). In the restful sleep group, HF-HRV was lower in the morning session relative to the evening session (t=4.11, p<.001; interaction: F(1,81)=21.20, p<.001, η_p^2=0.21). This effect was not observed in the sleep deprivation group (t=2.41, p=.074). N=1 participant was excluded from these analyses because their ECG data was not recorded in the morning session due to a technical fault.

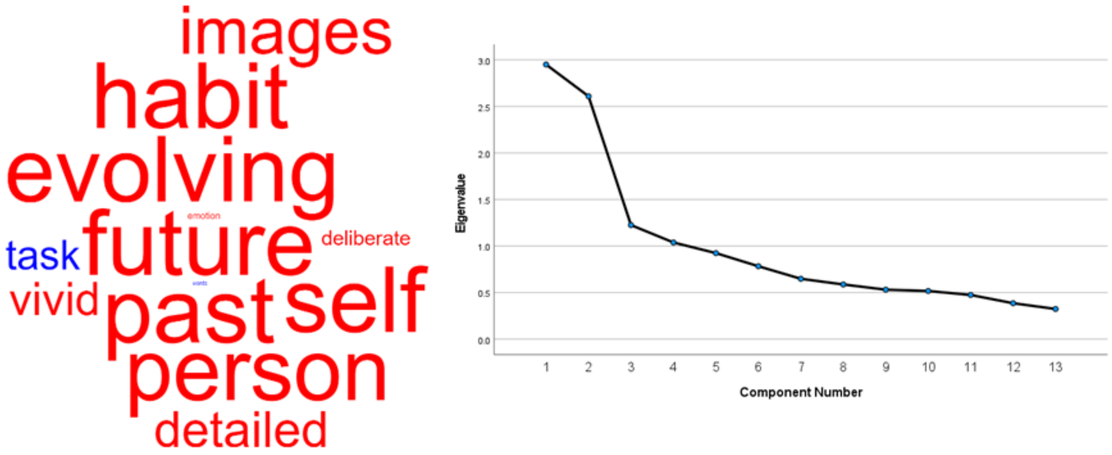

Supplementary Figure 3. Principal components analysis (PCA). Left: The loadings on the second component are presented as a word cloud. The colour of a word describes the direction of the relationship (red: positive, blue: negative) and the size of a word reflects the magnitude of the loading. Scores for this component were entered into a mixed-measures ANOVA with factors Task (0-back, 1-back), Session (Evening, Morning) and Group (Sleep Deprivation, Restful Sleep). There were no main effects of Task ($F(1,80)=0.81$, $p=.37$, $\eta_p^2=0.01$) or Group ($F(1,80)<0.01$, $p=.96$, $\eta_p^2<0.01$), but a main effect of Session ($F(1,80)=3.96$, $p=.05$, $\eta_p^2=0.05$) indicated that this thought pattern emerged to a greater extent in the morning than the evening. No significant interactions were observed for Task*Group ($F(1,80)=0.06$, $p=.81$, $\eta_p^2<0.01$), Session*Group ($F(1,80)=0.50$, $p=.48$, $\eta_p^2<0.01$), Task*Session ($F(1,80)=0.27$, $p=.60$, $\eta_p^2<0.01$) or Task*Session*Group ($F(1,80)=0.15$, $p=.70$, $\eta_p^2<0.01$). Right: Scree plot for the PCA.

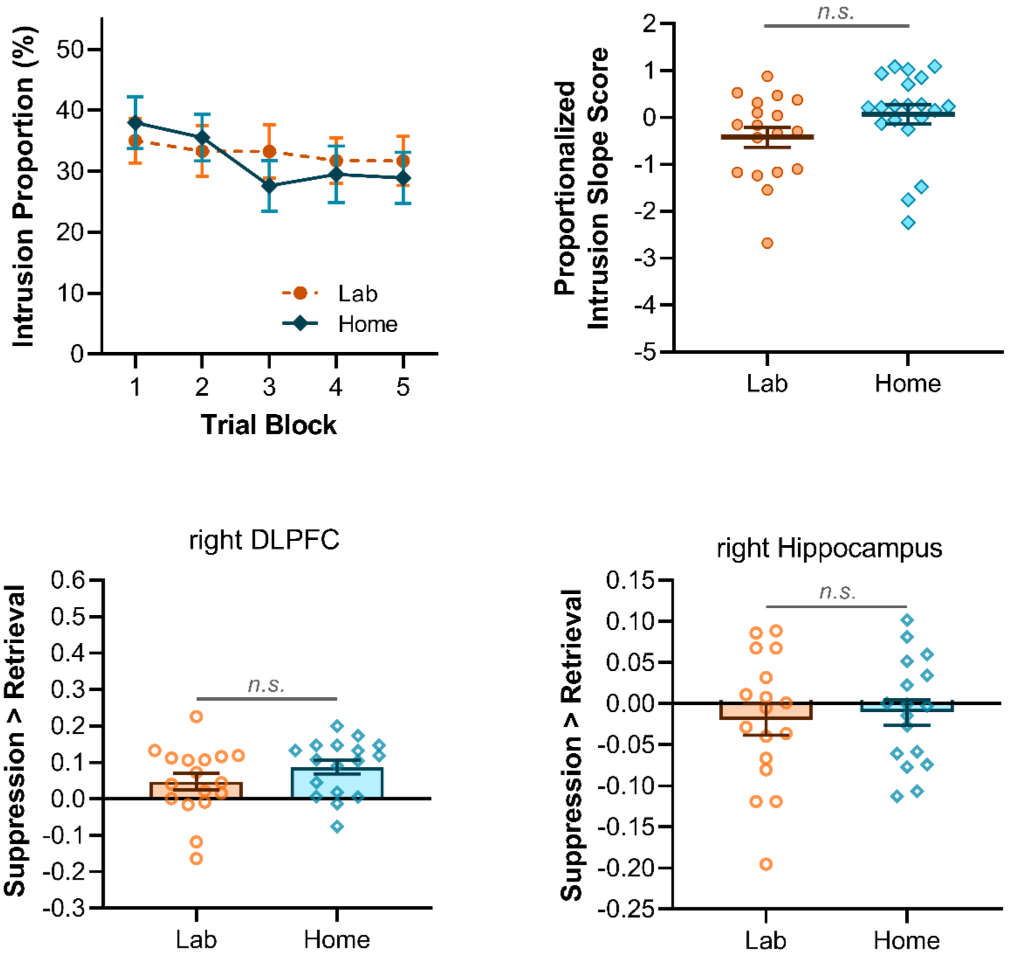

Supplementary Figure 4. The impact of in-lab as compared to at-home sleep deprivation on memory control. Top left: intrusion proportion scores from the sleep deprivation group were submitted to a mixed 2 (Location: Lab, Home) x 5 (Trial Block: 1, 2, 3, 4, 5) x 2 (Scene Valence: Negative, Neutral) ANOVA. There was a significant main effect of Trial Block ($F(3.11,111.85)=3.62$, $p=.014$, $\eta p2=0.09$, Greenhouse-Geisser corrected) and a significant interaction between the factors Scene Valence and Location ($F(1,36)=5.04$, $p=.031$, $\eta p2=0.12$ but no other significant main effects (both $p \geq .726$) or interactions (all $p \geq .113$). Top right: proportionalized intrusion slope scores were not affected by sleep deprivation location ($W=243$, $p=.067$, $rrb=0.35$). Bottom left and bottom right: activation in right dorsolateral prefrontal cortex (DLPFC; bottom left) and right hippocampus (bottom right) were analysed using mixed 2 (Location: Lab, Home) x 2 (Memory Process: Retrieval, Suppression) x 2 (Scene Valence: Negative, Neutral) ANOVAs. For rDLPFC, there was a significant main effect of Memory Process ($F(1,32)=21.84$, $p<.001$, $\eta p2=0.41$), but no other significant main effects (both $p \geq .247$) or interactions (all $p \geq .256$). For rHC, there were no significant main effects (all $p \geq .409$) or interactions (all $p \geq .467$). Plots illustrate suppression > retrieval contrasts.

Supplementary Table 1. Intrusion proportion (%), separately for each group and valence category

	Trial block				
	1	*2*	*3*	*4*	*5*
Sleep-deprived					
Negative	36.09 (3.48)	34.38 (3.30)	29.86 (3.45)	30.97 (3.33)	29.45 (3.26)
Neutral	37.05 (2.98)	34.56 (3.13)	30.73 (3.17)	30.16 (3.54)	31.02 (3.32)
Sleep-rested					
Negative	43.58 (3.81)	38.25 (3.87)	32.12 (3.69)	28.90 (3.42)	32.47 (3.73)
Neutral	47.22 (4.22)	39.73 (4.23)	33.31 (4.23)	32.49 (4.23)	28.82 (3.88)

Intrusion proportions are shown as means with SEM in parentheses.

Supplementary Table 2. Significant clusters found for seeds in the default mode network (DMN) and cognitive control network (CCN)

Seed	*X Y Z (MNI)*	*No. voxels*	*Activation*	*Region*
DMN	-44 -36 58	1958	Increase	Postcentral gyrus (left)
	8 -18 10	1601	Decrease	Thalamus (bilateral)
	56 -18 46	1504	Increase	Postcentral gyrus (right)
	56 -30 -28	816	Increase	Inferior temporal gyrus (right)
	-42 10 22	770	Increase	Inferior frontal gyrus (left)
	-44 -60 2	728	Increase	Lateral occipital cortex (left)
	-42 56 8	410	Increase	Frontal pole (left)
	48 16 28	318	Increase	Inferior frontal gyrus (right)
	6 42 -4	276	Decrease	Cingulate gyrus (anterior)
	-68 -30 12	229	Increase	Planum temporale (left)
	-36 -4 54	201	Increase	Precental gyrus (left)
	24 -2 66	163	Increase	Superior frontal gyrus (right)
CCN	-16 42 24	1084	Increase	Frontal pole (left)

Clusters showing a significant difference in connectivity for the contrast sleep deprivation>restful sleep. Results were thresholded using Gaussian Random Field theory, with a $p<0.001$ (uncorrected, two-sided) voxel threshold and an FDR-corrected cluster threshold at $p<0.05/7=0.007$ (to also correct for testing multiple seeds)**.**

Supplementary Table 3. Multidimensional experience sampling (MDES) thought probes

Dimension	***Question***	***1***	***10***
Task	My thoughts were focused on the task I was performing:	Not at all	Completely
Future	My thoughts involved future events:	Not at all	Completely
Past	My thoughts involved past events:	Not at all	Completely
Self	My thoughts involved myself:	Not at all	Completely
Person	My thoughts involved other people:	Not at all	Completely
Emotion	The emotion of my thoughts was:	Negative	Positive
Images	The contents of my thoughts were in the form of images:	Not at all	Completely
Words	The contents of my thoughts were in the form of words:	Not at all	Completely
Vivid	My thoughts were vivid as if I was there:	Not at all	Completely
Detailed	My thoughts were detailed and specific:	Not at all	Completely
Deliberate	My thoughts were:	Spontaneous	Deliberate
Habit	My thoughts had recurrent themes similar to those I have had before:	Not at all	Completely
Evolving	My thoughts tended to evolve in a series of steps:	Not at all	Completely

Supplementary Table 4. Proportion correct (%) in the n-back component of the morning MDES task, separately for each group and condition

	0-back	***1-back***
Sleep-deprived	90.70 (3.43)	96.51 (1.97)
Sleep-rested	91.03 (3.61)	92.31 (4.32)

A 2 (Group: Sleep Deprivation, Restful Sleep) x 2 (Condition: 0-Back, 1-Back) ANOVA on n-back identification accuracy revealed no significant main effect of Group ($F(1,80)=0.24$, $p=.625$, $\eta_p^2<0.01$) or Condition ($F(1,80)=1.70$, $p=.196$, $\eta_p^2=0.02$), and no significant interaction between factors ($F(1,80)=0.69$, $p=.408$, $\eta_p^2=0.01$). Mean values are shown with SEM in parentheses.

Supplementary Table 5. Proportionalized affect suppression scores for negative scenes, separately for each group and memory process

	Memory process		
	Baseline	*Retrieve*	*Suppress*
Sleep-deprived	0.14 (0.04)	0.11 (0.03)	0.13 (0.05)
Sleep-rested	0.07 (0.04)	0.17 (0.06)	0.16 (0.07)

Affect ratings gathered during the affect evaluation tasks were used to measure overnight changes in subjective emotional reactivity to negative scenes. Mean affect ratings were calculated for each participant, session (evening, morning), and image condition (baseline, retrieve, suppress). Affect suppression scores were calculated by subtracting the averaged values in the evening session from those in the morning session. To account for individual differences in emotional responding in the evening session, affect suppression scores were divided by the mean affect rating at the evening session to produce proportionalized affect suppression scores. Greater scores reflect more positive affect evaluations in the morning session compared with the evening session. Proportionalized affect suppression scores were applied to a mixed 2 (Group: Sleep Deprivation, Restful Sleep) x 3 (Image Condition: Retrieve, Suppress, Baseline) ANOVA. The analysis revealed no significant main effects (Group: $F(1,72)=0.01$, $p=.92$, $\eta_p^2<0.01$; Image Condition: $F(1.70,122.39)=0.88$, $p=.40$, $\eta_p^2=0.01$, *Greenhouse-Geisser corrected*), and the interaction was not significant ($F(1.70,122.39)=2.66$, $p=.082$, $\eta_p^2=0.04$, *Greehouse-Geisser corrected*). Mean values are shown with SEM in parentheses.

Supplementary Survey 1. Post-experiment questionnaire.

The following questions relate to the main attention task which you participated in during this experiment (i.e. the five blocks of trials where you viewed faces in red or green frames). Please answer the questions as *accurately* and *honestly* as you can.

1. Please rate the extent to which you used each of the following strategies in order to keep the *pictures* from coming to mind when you were presented with *RED-framed faces*.

a) I simply moved my eyes away from the *RED-framed faces* so I didn't have to look at them.

Never	Rarely	Sometimes	Often	Always
0	1	2	3	4

b) Although I kept my eyes on the *RED-framed faces*, I covertly shifted my attention to a different spot on the screen/elsewhere in the room, so I could avoid looking at the *RED-framed faces.*

Never	Rarely	Sometimes	Often	Always
0	1	2	3	4

c) I shifted my attention to something else in my mind, such as another image, word, idea, sound, or memory. In other words, I came up with an alternative thought in order to prevent the *associated pictures* from coming to mind/distract myself.

Never	Rarely	Sometimes	Often	Always
0	1	2	3	4

d) I paid close attention to the visual details of the RED-framed faces on the screen to distract myself from thinking about the related picture.

Never	Rarely	Sometimes	Often	Always
0	1	2	3	4

e) I simply focused on blocking/pushing out thoughts of the *associated pictures*, without replacing them with any other thoughts.

Never	Rarely	Sometimes	Often	Always
0	1	2	3	4

f) Other, please describe your strategy below, after rating how often you employed it.

Never	Rarely	Sometimes	Often	Always
0	1	2	3	4

____

____

2. Sometimes people suspect that their memory will be tested later on *associated pictures* for *faces* presented in a RED frame, even though they have been asked to not think about these *associated pictures*. Each of the following three statements is intended to measure whether you ever INTENTIONALLY made an effort to think about the pictures for the *RED-framed faces* (so please only consider those instances in which you purposefully thought of a response, not those in which a response automatically came to mind). Please make a rating for each statement and be as honest as possible with your ratings.

	Never	Rarely	Sometimes	Frequently	Very Frequently
a) When I saw a *RED framed face*, I quickly checked to see if I remembered the *associated picture*	0	1	2	3	4
b) After a *RED framed face* went off the screen I checked to see if I still remembered the *associated picture*	0	1	2	3	4
c) When I saw a *RED framed face*, I thought about the *picture* that went with it to improve my memory for that face-picture pair.	0	1	2	3	4

d) Please describe, in your own words, why you engaged in the above behaviours (a-c) if you circled anything other than 0 (never).

____

____

____

3. On *GREEN framed face* trials:

a) How often did you think of a verbal description of the *associated* picture?

Never	Rarely	Sometimes	Often	Always
0	1	2	3	4

b) Did you ever mistakenly not think about the *associated picture*?

Never	Rarely	Sometimes	Often	Always
0	1	2	3	4

c) How often did you try your best to think of the **entire**, **complete** *associated picture* (as opposed to only a part of it)?

Never	Rarely	Sometimes	Often	Always
0	1	2	3	4

d) How **vividly** would you say you were able to visualize the pictures (please circle one option)?

1. Perfectly clear and as vivid as normal vision
2. Clear and reasonably vivid
3. Moderately clear and vivid
4. Vague and dim
5. No image at all, you only “knew” that you were thinking of the picture

4. Please rate how frequently the UNPLEASANT pictures associated with the *RED-framed faces* came to mind at the following times during the main phase of the experiment:

a) When the face originally linked with the picture appeared on the screen

Never	Rarely	Sometimes	Often	Always
0	1	2	3	4

b) When making the 1-3 decision about how frequently the item came to mind during the trial

Never	Rarely	Sometimes	Often	Always
0	1	2	3	4

c) In between the trials (i.e., when the white cross was on the screen)

Never	Rarely	Sometimes	Often	Always
0	1	2	3	4

d) During the short breaks

Never	Rarely	Sometimes	Often	Always
0	1	2	3	4

e) When other faces (i.e., those NOT originally associated with the picture) were on the screen

Never	Rarely	Sometimes	Often	Always
0	1	2	3	4

5. Please rate how frequently the NEUTRAL pictures associated with the *RED-framed faces* came to mind at the following times during the main phase of the experiment:

a) When the face originally linked with the picture appeared on the screen

Never	Rarely	Sometimes	Often	Always
0	1	2	3	4

b) When making the 1-3 decision about how frequently the item came to mind during the trial

Never	Rarely	Sometimes	Often	Always
0	1	2	3	4

c) In between the trials (i.e., when the white cross was on the screen)

Never	Rarely	Sometimes	Often	Always
0	1	2	3	4

d) During the short breaks

Never	Rarely	Sometimes	Often	Always
0	1	2	3	4

e) When other faces (i.e., those NOT originally associated with the picture) were on the screen

Never	Rarely	Sometimes	Often	Always
0	1	2	3	4

6. During the main phase of the experiment, you were asked to bring to mind and focus on the pictures associated with the faces in *green frames*. Despite these instructions, some people intentionally fail to bring these faces to mind. Please rate how frequently you intentionally failed to think about the picture associated with a green-framed face, if it happened to be a:

a) *Unpleasant picture:*

Never	Rarely	Sometimes	Often	Always
0	1	2	3	4

b) *Neutral picture:*

Never	Rarely	Sometimes	Often	Always
0	1	2	3	4

7. The main phase in this experiment is long and requires quite a bit of focus and energy. Overall, how much effort did you feel the main task took?

None		Medium amount		Great deal
0	1	2	3	4

8. To what extent do you feel that you completed the main phase of this experiment as required?

None		Medium amount		Great deal
0	1	2	3	4

9. Overall, how much motivation did you have to complete the main phase of the experiment properly?

None		Medium amount		Great deal
0	1	2	3	4

10. How well were you able to tell apart the faces in this experiment?

Not At All		Somewhat		Very Well
0	1	2	3	4

11. Please describe what strategy you used for preventing yourself from visualizing **pictures** associated with RED-framed faces:

____

____

11b) Did this strategy differ at all for any clusters/categories of pictures? If so, please describe how so and also what clusters/categories of pictures you noticed.

____

____

____

____

12. During the main phase of the experiment, you were also asked to rate how often a picture associated with each face came to mind on a three-point scale <u>after</u> each trial. How often did you think about what button you would press before the rating scale appeared (i.e., when the face was still on the screen)?

a) For the *RED*-framed faces:

Never	Rarely	Sometimes	Often	Always
0	1	2	3	4

b) For the *GREEN*-framed faces:

Never	Rarely	Sometimes	Often	Always
0	1	2	3	4

13. This question pertains to the ratings you made during the main phase of the experiment, where you were supposed to report (with a 1-3 rating) the extent to which you thought about the associated picture for the face that was just presented. Specifically, we're interested in those trials in which a face was presented with a *red frame*—those trials where you were supposed to *not think* of the associated picture. For those trials, please rate the extent to which you think having to make these ratings ***improved*** your memory for the associated pictures for:

a. *Unpleasant pictures:*

None		Medium amount		Great deal
0	1	2	3	4

b. *Neutral pictures:*

None		Medium amount		Great deal
0	1	2	3	4

14. In the first session, you were presented with each of the faces and we asked if you could visualize and recall the associated picture. How often did you say that you could recall the image when you could not actually visualize it (i.e. you relied on recognizing the picture when presented with the three options afterwards)?

a) *Unpleasant picture:*

Never	Rarely	Sometimes	Often	Always
0	1	2	3	4

b) *Neutral picture:*

Never	Rarely	Sometimes	Often	Always
0	1	2	3	4
